# Diverse Short-Term Dynamics of Inhibitory Synapses Converging on Striatal Projection Neurons: Differential Changes in a Rodent Model of Parkinson's Disease

**DOI:** 10.1155/2015/573543

**Published:** 2015-06-08

**Authors:** Janet Barroso-Flores, Marco A. Herrera-Valdez, Violeta Gisselle Lopez-Huerta, Elvira Galarraga, José Bargas

**Affiliations:** ^1^División de Neurociencias, Instituto de Fisiología Celular, Universidad Nacional Autónoma de México, 04510 Ciudad de México, Mexico; ^2^Departamento de Matemáticas, Facultad de Ciencias, Universidad Nacional Autónoma de México, 04510 Ciudad de México, Mexico

## Abstract

Most neurons in the striatum are projection neurons (SPNs) which make synapses with each other within distances of approximately 100 *µ*m. About 5% of striatal neurons are GABAergic interneurons whose axons expand hundreds of microns. Short-term synaptic plasticity (STSP) between fast-spiking (FS) interneurons and SPNs and between SPNs has been described with electrophysiological and optogenetic techniques. It is difficult to obtain pair recordings from some classes of interneurons and due to limitations of actual techniques, no other types of STSP have been described on SPNs. Diverse STSPs may reflect differences in presynaptic release machineries. Therefore, we focused the present work on answering two questions: Are there different identifiable classes of STSP between GABAergic synapses on SPNs? And, if so, are synapses exhibiting different classes of STSP differentially affected by dopamine depletion? Whole-cell voltage-clamp recordings on SPNs revealed three classes of STSPs: depressing, facilitating, and biphasic (facilitating-depressing), in response to stimulation trains at 20 Hz, in a constant ionic environment. We then used the 6-hydroxydopamine (6-OHDA) rodent model of Parkinson's disease to show that synapses with different STSPs are differentially affected by dopamine depletion. We propose a general model of STSP that fits all the dynamics found in our recordings.

## 1. Introduction

Short-term synaptic plasticity (STSP) is a form of information processing in neuronal circuits [1–3]. A consensus has been reached where it mainly depends on the interaction between Ca^2+^ channels and Ca^2+^ management and the release machinery of the synaptic terminals [3–10]. Postsynaptic targets and local modulation also have a role [11–14]. Nevertheless, under the same stimulation protocol, temperature, postsynaptic targets, and ionic and modulatory environments, different classes of presynaptic neurons display particular forms of STSP [3, 10, 15, 16]. As a result, the variety of postsynaptically observed STSPs suggests a diversity of presynaptic sources [2, 7, 11, 14–20] and gives reason to suspect that there can be different classes of synaptic entries on to the same postsynaptic target [5–7].

However, there is no knowledge about the variety of STSP dynamics displayed by the synaptic GABAergic entries that target SPNs. One reason may be the difficulty to obtain paired recordings from some classes of interneurons and SPNs (e.g., [21]). Perhaps, due to these limitations, the existence of functionally relevant synaptic connections from interneurons on to SPNs in control conditions has been questioned [22]. Another reason may be the inherent limitations of actual techniques, including optogenetics (e.g., [23]).

We used field stimulation at 1 mm distance from the recorded cell to select long range connections and show that, after stimulus trains at 20 Hz, samples that showed three qualitatively different classes of STSP could be disclosed by recording SPNs: short-term depression (STD), short-term facilitation (STF), and a combination of both following biphasic dynamics (STB) [3, 24]. None of these forms of STSP were similar to STSP obtained from synapses between SPNs under the similar conditions (STD) (cf. [20, 25, 26]). Moreover, synapses that exhibit these various forms of STSP were differentially affected by dopamine (DA) depletion [27] in the 6-OHDA rodent model of Parkinson's disease. These results suggest that, as in other brain nuclei, GABAergic inputs from probably different classes of GABAergic neurons make synapses on to SPNs. In addition, we propose a novel general model that describes common principles underlying all these types of STSP dynamics in control conditions and also gives insights into the dynamics observed in pathological conditions. The modeling suggests that DA loss results in substantial changes in the presynaptic machinery of some GABAergic neurons.

## 2. Methods

### 2.1. Animals

Male Wistar rats of about 25 postnatal days from our Animal House were used in most experiments. Male BAC-TH-GFP transgenic mice were used in some experiments. Because the results were similar, we will discuss the results obtained from rats. The number of animals used in the experimental samples was the minimum possible to attain statistical significance. Animal suffering was avoided. All the procedures followed the guidelines for the use of animals in biomedical experiments provided by the National University of Mexico and the National Institutes of Health.

### 2.2. 6-OHDA Lesions

Animals were deeply anesthetized with a mixture of ketamine (85 mg/kg, i.p.) and xylazine (15 mg/kg, i.p) and immobilized on a stereotactic frame to receive a unilateral injection of 6-OHDA (8 *μ*g in 0.2 *μ*L with 0.2 mg/mL ascorbic acid) at 0.1 *μ*L/min in the substantia* nigra pars compacta* (coordinates 3.8 mm caudal, 1.8 mm lateral to bregma, and 7.1 mm ventral to the skull surface). The syringe was left in place for 10 minutes to allow diffusion and maximize tissue retention of the solution.

### 2.3. Rotational Behavior

Success of the lesion was evaluated by testing turning behavior 7 days after the surgery. The protocol used for the assessment was similar to that described before for neonatal and adult lesioned rats [28–31]. Rats were placed in automated rotometer bowls. After acclimatization, amphetamine (4 mg/kg, i.p.) was administered; left and right full body turns were recorded for 90 minutes by a home-made computerized monitor system. Animals showing >500 ipsilateral turns toward the lesioned side were considered for further experiments.* In vitro* recordings from slices were obtained after 3–8 days after the behavioral test.

### 2.4. Tyrosine Hydroxylase Immunoreactivity

The degree of lesion was also verified using tyrosine hydroxylase (TH) immunostaining in a subset of rats. One week after rotational behavior, rats were perfused transcardially with 4% paraformaldehyde in 0.1 M phosphate-buffered saline (PBS), pH = 7.3. Coronal slices (40 *μ*m) were obtained and incubated with 1% bovine albumin to block nonspecific binding sites and for 36 h with a rabbit polyclonal antibody against TH (1 : 500 Millipore) dissolved in PBS containing 0.25% Triton-X. The slices were then rinsed thrice with PBS and incubated with a goat versus rabbit secondary antibody during 1 h. This antibody was conjugated with FITC (FI-1000, Vector Laboratories, Burlingame, CA). Samples were mounted with vectashield (Vector Laboratories, Burlingame, CA) and observed in a confocal microscope Olympus FV-1000. 338 XY images were obtained (320 × 320 pixels) using a motorized stage (prior scientific H117) with a 20x oil immersion objective, stimulating at 488 nm of monochromatic light. Image analysis was performed using Software FluoView 1000.

### 2.5. Brain Slices

Animals were deeply anaesthetized with isoflurane and transcardially perfused with 5–7 mL ice-cold solution containing (in mM) 225 sucrose, 7 KCl, 1 MgCl_2_, 0.5 CaCl_2_, 28 NaHCO_3_, and 10 glucose, 1 ascorbic acid, and 3 pyruvate (pH 7.2 with NaOH; saturated with 95% O_2_ and 5% CO_2_). Then, rat brains were removed into ice-cold saline. Sagittal slices (250 *μ*m) were obtained and stored for recuperation 30 minutes in saline solution containing (in mM) 126 NaCl, 3 KCl, 1 MgCl_2_, 2.0 CaCl_2_, 25 NaHCO_3_, and 10 glucose (pH 7.4 with NaOH, 298 mOsM/L with glucose; saturated with 95% CO_2_ and 5% O_2_). Slices were transferred to a custom Plexiglas recording chamber and were continuously superfused with oxygenated saline (3 mL/min). Individual neurons were visualized (40x water immersion objective) under differential interference contrast (DIC) enhanced visual guidance using infrared videomicroscopy.

### 2.6. Electrophysiological Recording

Whole-cell patch-clamp recordings were performed with micropipettes made of borosilicate glass, fire-polished for D.C. resistances of about 3–6 MΩ. Internal solution had a high Cl^−^ concentration of 40 mM, while extracellular concentration was 135 mM for a theoretical equilibrium potential for chloride of 0 mV, enough to have inhibitory postsynaptic currents (IPSCs) as inward currents at −80 mV holding potential (in mM): 72 KH_2_PO_4_, 36 KCl, 2 MgCl_2_, 10 HEPES, 1.1 EGTA, 0.2 Na_2_ATP, 0.2 Na_3_GTP, 5 QX-314, and 0.5% biocytin (pH = 7.2, 275 MOsm/L [32]). QX-314 prevented action potentials from occurring and allowed for stable voltage-clamp recording at different holding potentials.

Synaptic events were evoked with field stimulation with the use of a pencil bipolar concentric tungsten electrode (12 *μ*m at the tip; 20–90 kΩ in the tissue). Trains of 10 shocks (50 ms of interstimulus interval; 0.2–0.4 ms duration; 1–90 V delivered through the stimulating electrode; at a frequency of 0.1 Hz) were controlled with a computer interface (see below) and isolation units (Digitimer LTD, Hertfordshire, UK) between the computer and the stimulating electrodes to quickly adjust stimulus parameters during the experiment. The field electrode was positioned in the dorsal striatum. The distance between recording and stimulating electrode in all configurations was about 1000 *μ*m. All experiments were realized in the presence of 6-cyano-2,3-dihydroxy-7-nitro-quinoxaline disodium salt (CNQX) (10 *μ*M) and D-(-)-2-amino-5-phosphonovaleric acid (APV) (50 *μ*M) to block ionotropic glutamatergic transmission.

The traces shown are the averages of 5 min recordings (25 traces). A small hyperpolarizing voltage command (10 mV) was constantly given during the experiment to monitor input conductance. Whole-cell access resistances were in the range 5–20 MΩ. Access resistance was continuously monitored and experiments were abandoned if changes >25% were encountered. No cell capacitance, series resistance, or liquid junction potential (2 mV) compensations were made. All recordings were filtered at 1–3 KHz and digitized with an AT-MIO-16E10 (National Ins., Austin TX) DAQ (NI-DAQ) board in a PC clone. Online data acquisition used custom programs made in the LabView environment (National Ins.). The NI-DAQ board was used to save the data on binary files in the computer hard disk for further offline analysis.

Digitized data saved on disk was imported for analysis and graphing into commercial software (Origin v.7. Microcal, Northampton, MA, and MatLab). IPSCs amplitudes were measured from basal line to peak from the first response (IPSC_1_). The STSP curves were graphed as the amplitude of the* n*th IPSC normalized by the amplitude of the first IPSC (IPSC_1_) and fitted to a generic multiplication of exponential functions after normalization:

(1) $$\begin{array}{c} \frac{{IPSC}_{n}}{{IPSC}_{1}}=\left(\;A-e^{-x/\tau_{Rec}}\;\right)*\left(\;e^{-x/\tau_{Dep}}+B\;\right)+C. \end{array}$$

Exponentials functions correspond to facilitating and depressing plasticity, respectively [33], because both processes appear to occur at the same time in any synapse [3].* A* represents the initial steady state value that the recovery process tends to approach.* B* represents the initial value of the depletion process and* C* is a balancing parameter that helps the fitting procedure (Levenberg-Marquardt) to begin with a value = 1. In this way, all experimental and normalized IPSCs average amplitudes could be fitted. Thus, these parameters are nondimensional except for the time constants *τ*_Rec_ and *τ*_Dep_ that denote the time constant for recovery (e.g., vesicle replenishment) and depleting processes, respectively [10]. Values obtained could or not give a hint for the initial parameters for the model. Intensity-amplitude functions of the synaptic responses (*I*-*A* plots) were fitted to

(2) $$\begin{array}{c} A\left(\;I\;\right)=\frac{A_{max}}{1+e^{\left(-k\left(I-I_{h}\right)\right)}}, \end{array}$$

where *A*_max_ denotes maximal amplitude of evoked IPSC, *I* denote stimulus intensity normalized to threshold units, *I*_*h*_ denotes the stimulus intensity that evokes IPSCs of half-maximal amplitude, and *k* denotes a slope factor (that can be thought of as proportional to the number of terminals recruited as a function of stimulus intensity [34]).

### 2.7. A Model of Short-Term Synaptic Plasticity

Here, we propose a computational model that reproduces the different types of STSP dynamics in control conditions and approaches the experimental data after DA depletion. Even though the model does not completely fit all the DA depletion cases, the results illustrate the processes that may be changed after DA loss. The model uses two dynamic variables that comprise the many mechanisms involved: occupancy of vesicles in the readily releasable pool (RRP, *x*(*t*)) and the probability of release (*p*(*t*)). Assuming a presynaptic neuron fires a train of action potentials at times *t*_*k*_, *k* = 0,1, 2,3…, the release dynamics of the presynaptic terminal can be described by

(3) dxtdt=xx∞−xtτRec−px∑ϕt−tk,dptdt=pp∞−ptτDep+1−p∑qhkϕt−tk,

where

(4) $$\begin{array}{c} \phi \left(\;t,\tau_{\phi },A\;\right)=A\frac{t}{\tau_{\phi }}e^{\left(1-t/\tau_{\phi }\right)} \end{array}$$

models the shape of a pulse in the presynaptic terminals or afferent volley. For simplicity, we used *A* = *τ*_*ϕ*_ = 1 and $\;q\left(h\right)=\overset{-}{q}he^{1-h/\tau_{Dep}}$.

The parameters *x*_*∞*_ and *p*_*∞*_ represent the steady states of the occupancy of the readily releasable pool and the probability of release, respectively. *τ*_Rec_, *τ*_Dep_ represent the recovery and depletion time constants, respectively. An increase in *p*(*t*), denoted by *q*(*h*), can be assumed to depend on the presynaptic interspike intervals, *h*, with *q* representing the maximum increase in the probability of release. In agreement with experiments, we used trains of 20 Hz with regular interspike intervals. All simulations were performed in python (https://www.python.org/) with the modules scipy (http://scipy.org/) and matplotlib (http://matplotlib.org/).

### 2.8. Drugs

6-Cyano-2,3-dihydroxy-7-nitro-quinoxaline disodium salt (CNQX), D-(-)-2-amino-5-phosphonovaleric acid (APV), QX-314, and bicuculline (Sigma-Aldrich-RBI, St. Louis, MO, USA) stock solutions were freshly prepared and added to the superfusion during each experiment to obtain the required final concentration.

## 3. Results and Discussion

### 3.1. Repertoire of Short-Term Synaptic Plasticities in GABAergic Synapses That Target Striatal Projection Neurons

**Figure 1:**
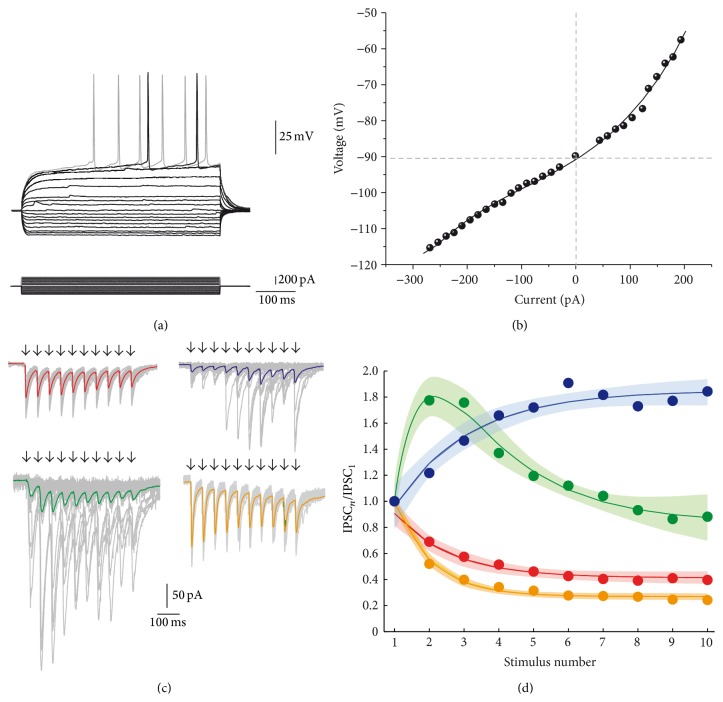
Different classes of short-term synaptic plasticity from inhibitory synapses that target striatal projection neurons. (a) Recorded neurons are striatal projection neurons or spiny neurons. Top: depolarizing and hyperpolarizing voltage responses. Bottom: intracellular current steps that evoked the voltage responses. (b) Current-voltage relationship (*I-V* plot) measured from the traces in* A*. (c) Protocol to evoke short-term synaptic plasticity (STSP): trains of intrastriatal field stimulus at 20 Hz are pointed with arrows. Thick colored traces represent the average of 25 individual responses and thin gray traces represent individual responses showing quantal variation. Trains of inhibitory postsynaptic currents (IPSCs) were evoked equally in all cases but the class of STSP obtained varied: red traces: short-term synaptic depression (STD); blue traces: short-term synaptic facilitation (STF); green traces: short-term biphasic plasticity (STB). For comparison, yellow traces are the response to antidromic field stimulation from the globus pallidus (STD) which mostly represents connections between SPNs (López-Huerta et al., 2013 [28]). Basal current is subtracted to measure each individual IPSC. (d) STSP dynamics of averaged and normalized IPSCs from samples of each class of STSP. Colored circles represent average experimental data. Thick colored lines represent the fit of (1): IPSC_*n*_/IPSC_1_ = (*A* − *e* ^−*x*/*τ*_Rec_^)*∗*(*e* ^−*x*/*τ*_Dep_^ + *B*) + *C*. The surrounding shadow area denotes estimation error. Note that three classes of STSP are present.

The diversity of striatal GABAergic interneurons is large [35]. An intrastriatal field electrode may stimulate any of them. In the present case, field stimulation was performed 1 mm away from the recorded SPN (see Section 2) since inhibition between striatal projection neurons (SPNs) is restricted to about 100 *μ*m. SPNs were identified by their typical voltage responses to intracellular current injections (Figure 1(a)) which showed inward rectification and prolonged latency to fire the first action potential. Current-voltage relationships (*I-V* plots) were nonlinear (Figure 1(b)). To evoke inhibitory inputs on to SPNs, 20 Hz trains of 10 field stimuli were delivered every ten seconds (0.1 Hz) in the presence of glutamate receptor blockers (10 *μ*M CNQX plus 50 *μ*M APV) (arrows in panels of Figure 1(c)). Evoked IPSCs recorded on SPNs (Figure 1(c)) exhibited three classes of STSP (representative experiments): depressing (STD; red, top left; *n* = 6), facilitating (STF; blue, top right; *n* = 6), and a combination of facilitation and depression or biphasic (STB; green, bottom left; *n* = 6). Thick colored traces represent the average of amplitudes from representative experiments. Thin gray traces represent individual responses to illustrate intrinsic quantal variability of evoked discrete IPSCs. Both facilitating and biphasic dynamics exhibited failures (Figure 1(c)), suggesting presynaptic elements with initial low probability of release. Depressing synapses almost had no failures, suggesting an initial high probability of release. From previous work [20, 25, 26, 28, 31, 36, 37], STSP of connections among SPNs is known to be depressing and with a higher depression rate than that shown between fast-spiking (FS) interneurons and SPNs (shown in Figure 1(c) yellow, bottom right) [20, 25]. Indeed, the short-term dynamics of synapses between SPNs is different than those for synapses reported here. The depressing responses we found (Figure 1(c) top left, red) were not significantly different than those reported previously for FS-SPN connections [20] under the same conditions. To our knowledge, the other classes of STSP dynamics have not been reported in the striatum.

One source of variance of these responses is the intrinsic quantal variability of discrete synaptic events that precludes the use of parametric statistics for comparing samples quantitatively (see grey traces in Figure 1(c)). In addition, many different neuron types have been described in the striatum [21, 35, 38–43] and several of them may exhibit similar classes of STSP, which may be another source of variation. Finally, some contamination when using field electrodes cannot be discarded. Therefore, as a first step, our goal was to obtain a clear categorization of the classes of STSP exhibited by the GABAergic inputs converging on to SPNs without focusing on the types of presynaptic elements that may cause them. To accomplish this goal, we averaged samples of experiments with the same dynamics, STD, STF, and STB, and then fitted (1) to these averages (Figure 1(d), thick colored lines, estimation errors shown as shaded areas around the fit). This procedure clearly distinguished between three different classes of short-term dynamics. In this way, preliminary quantitative measurements of obvious qualitative differences were obtained (Table 1). STD (red), STF (blue), and STB (green) were evoked maintaining the same cellular target: SPNs, stimulus trains, and extracellular media. Once evoked, the dynamics were robust. Therefore, these results suggest that different classes of inhibitory synapses exhibiting various STSP dynamics make contact with SPNs as suggested here and previously [20, 21, 35, 38, 39, 41–43]. Equation (1) (Section 2) fitted all classes of STSP. In synapses exhibiting STD, the time constant for recovery is > 6 times slower than the time constant for depletion (*τ*_Rec_ for STD versus *τ*_Dep_ for STD; *P* < 0.05, the Friedman test and the* post hoc* Dunn test) which explains why depression is the dominant process. In contrast, for STF synapses, the time constant for recovery almost doubles the time constant for depletion (*τ*_Rec_ for STF versus *τ*_Dep_ for STF; *P* > 0.05, the Friedman test and the* post hoc* Dunn test) resulting in a facilitation process. In biphasic synapses, both processes were slower but the depletion process was approximately 50% slower (*τ*_Rec_ for STB versus *τ*_Dep_ for STB; *P* < 0.05, the Friedman test and the* post hoc* Dunn test); after an initial facilitation, depression dominated the last part of the dynamics. In summary, in facilitating and biphasic synapses, the depleting process tends to be slower than in depressing synapses, while the probability of release is higher in depressing synapses. It would be improbable that a single class of presynaptic element possesses all these different mechanisms while contacting the same postsynaptic targets, under the same conditions. Nevertheless, we linked another set of parameters to the present one to better distinguish between different classes of GABAergic entries on to SPNs.

### 3.2. The IPSCs Amplitudes and the Rate of Synapse Recruitment Differ in Synapses Exhibiting Different Classes of STSP

**Figure 2:**
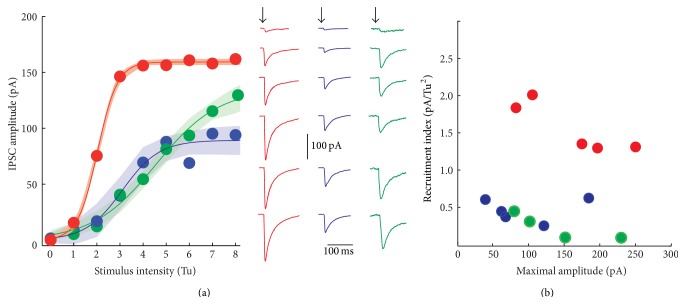
Intensity-amplitude relationships. (a) Intensity-amplitude relationships (*I*-*A* plots) for average synaptic amplitudes belonging to each of the STSP classes. Each dot represents an average from experimental samples. Thin lines are the fits for: IPSCAmp(*I*) = *A*_max_/(1 + *e* − *k*(*I* − *I*_*h*_)). The colored shadowed areas around the fit show the estimation errors as a function of threshold units strength. Right: fitted curves again showed differences among each other. In agreement with color: representative recordings from each type of* I*-*A* plot. Arrows point to the time of stimulus. (b) Recruitment index (slope factor divided by the stimulus necessary to attain half-maximal amplitude, *k*/*I*_*h*_) as a function of maximal amplitude reached by the IPSCs. The red plot and dots correspond to synapses exhibiting STD; the blue plot and dots correspond to those synapses showing STF; the green plot and dots correspond to synapses exhibiting STB. Depressing connections more easily recruit release sites as a function of stimulus strength.

Intensity-amplitude relationships (*I*-*A* plots) of synapses exhibiting different classes of STSP were explored. First, we determined the minimal stimulus that produces an IPSC response on a SPN. This stimulus was set as a threshold unit (Tu) in order to normalize all responses. Then, gradual increases in stimulus strength, measured in threshold units, evoked IPSCs with larger amplitudes.* I*-*A* experiments were fitted by a three-parameter sigmoidal function (see (2) in Section 2), where *A*_max_ denoted maximal amplitude of IPSC, *I*_*h*_ denotes the stimulus intensity that evokes IPSCs of half-maximal amplitude, and *k* is the slope factor proportional to the recruitment of release sites as a function of stimulus strength (Figure 2(a)). Again, due to intrinsic quantal variability we fitted the average of each sample (colored) in agreement with the STSP they were associated with, ± the estimation errors of the fits, illustrated as shaded areas (three different sigmoidal functions in Figure 2(a) and Table 2). STD synapses reached the saturation current faster than the other classes of synapses. There were significant differences between the slope factor (*k*) of STD synapses and the other classes of synapses (*P* < 0.05, Kruskal-Wallis with* post hoc* Dunn test) but not between STF and STB synapses. STF and STB recruited release sites at a lower rate (Figure 2(a); Table 2). A recruitment index (RI) was assessed by dividing the slope factor versus the stimulus necessary to attain half-maximal amplitude *k*/*I*_*h*_ and then plotted as a function of maximal amplitude (Figure 2(b)). STF and STB had this index < 1, while depressing synapses show an index > 1 (*P* < 0.05 between STD and STF; Table 2). In agreement with previous evidence [20, 25, 41], STD synapses may correspond to parvalbumin positive (PV+) cells or fast-spiking (FS) neurons whose axons extend radially and profusely. Maximal IPSC amplitudes were larger in STD compared to STF synapses (*P* < 0.05, Kruskal-Wallis with* post hoc *Dunn test) but not between them and STB synapses. Failures and heterogeneous release probability account for small amplitude averages in STF and STB synapses, given the high amplitudes that some individual traces may reach (Figure 1(c), grey thin traces). Pair recordings between FS and SPNs have been done [20, 25, 44, 45]. In contrast, not enough samples of pair recordings have been published between other interneuron classes and SPNs in the striatum [21]; the reason may be that their axons project to any destination from the soma into a round sphere of >1000 *μ*m radius (e.g. [21, 42]; the striatum has no layers or columns) making it hard to foretell where the connections are going to be in the space surrounding the recorded cell. However, these results suggest that, as in other brain circuits, many types of STSP may result from a diversity of GABAergic inputs on to SPNs [2, 7, 11, 14–20].

It is known that the amplitude of the synaptic events of some, but not all, GABAergic neurons or terminals in the striatum on to SPNs changes when tissue dopamine is lost [27]. Therefore, we may observe differential changes in evoked synaptic responses in synapses with different classes of STSPs after dopamine depletion.

### 3.3. Inputs That Display Distinct STSP Dynamics Are Affected Differentially by Dopamine Depletion

**Figure 3:**
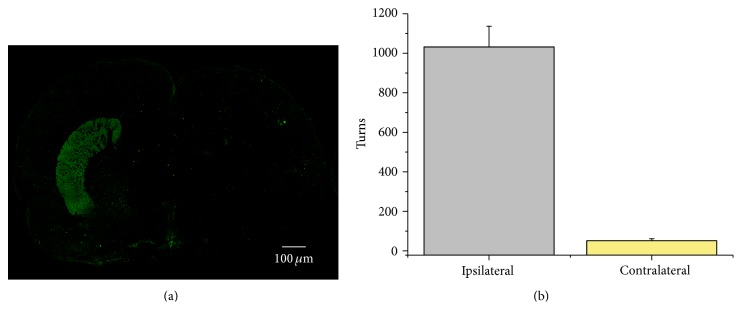
The 6-OHDA model of rodent Parkinsonism. (a) Coronal slice of a 6-OHDA injured rat. Green in the left side shows immunoreactivity to tyrosine hydroxylase (TH+), while the other side is virtually empty of TH+ fibers. In the case of mice, we used TH BAC GFP animals. (b) Histogram showing the number of body turns toward ipsilateral or contralateral sides after amphetamine injection (subcutaneous).

To examine how GABAergic inputs on to SPNs may be altered by dopamine loss, we used the 6-hydroxydopamine (6-OHDA) model of rodent hemi-Parkinsonism [28, 46]. 6-OHDA was injected (Figure 3) in the substantia nigra pars compacta (SNc) to eliminate dopamine (DA) afferents to the striatum. The success of lesion induced by 6-OHDA was tested evoking turning behavior with amphetamine 7 days after surgery (PD, 22–30). Some animals were used to observe the loss of dopamine fibers in the injured side (Figure 3(a)). The number of ipsilateral turns after amphetamine was significantly different compared to those towards the contralateral side (Figure 3(b)).

**Figure 4:**
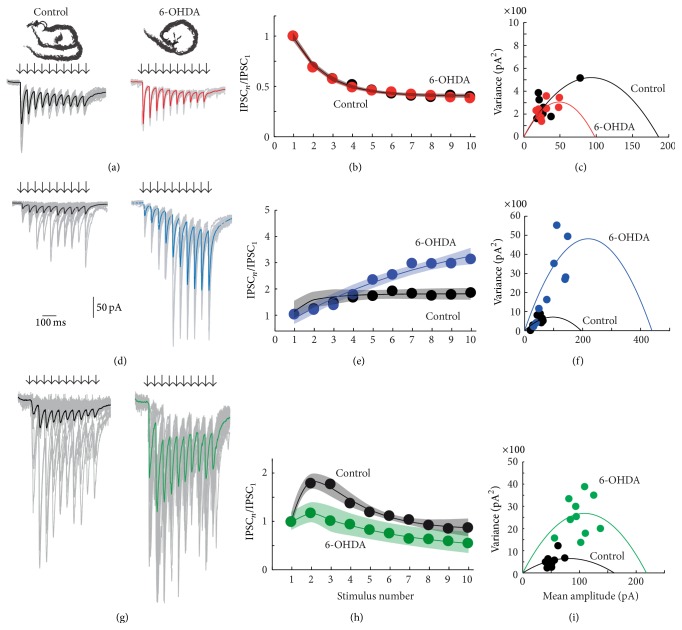
Synapses of 6-OHDA lesioned animals were affected differentially depending of the type of STSP that they expressed. (a) An STD synapse in control and after dopamine (DA) depletion (red). In these and next recordings, black or colored lines are the average of individual traces shown in grey. (b) Average short-term dynamics of both samples of neurons were similar (almost superimposable). (c) Variance-mean analysis of representative synapse in* A*. Except for one data point from the control, clouds of red and black dots are superimposable. (d) An STF synapse in control (black) and after DA depletion (blue). (e) Average short-term dynamics of both samples of neurons were different. After DA depletion short-term dynamics show more facilitation (blue) and failures decreased. Mean amplitude was larger. (f) Variance-mean analysis of representative synapse in (d). Control (black) and DA depleted (blue) data points distribute differently. Analysis showed that both probability of release and number of release sites probably increased. (g) An STB synapse in control (black) and after DA depletion (green). (h) Average short-term dynamics of samples of these synapses were different. After DA depletion short-term dynamics show less facilitation (green) and failures decreased. Mean amplitude was larger. (g) Variance-mean analysis of representative synapse in* A*. Control (black) and DA depleted (green) data points distribute differently. Analysis showed that both probability of release and number of release sites probably increased. Illustrated in Figure 6.

Evoked trains of IPSCs with predominant STD dynamics in controls (Figure 4(a) top left, black traces) and in 6-OHDA lesioned animals did not differ significantly (Figure 4(a) top right, red traces). In fact, the depression dynamics in both samples were very similar (Figure 4(b)) even when their time constants for recovery and depletion, as fitted, were slightly different (Table 1). The quantal variance (grey thin traces in Figure 4(a)) as a function of IPSC amplitude, in both control and lesioned preparations, was similarly distributed (except for one data point; Figure 4(c); NS), indicating that the probability of release and the number of release sites were not significantly affected by dopamine deprivation [47] in STD synapses (NS).

In contrast, synapses with predominant STF profiles (Figures 4(d)–4(f)) were greatly affected by dopamine loss. Dopamine depletion increased the apparent or weighted release probability (Pw [47]) from (mean ± SEM) 0.23 ± 0.03 to 0.42 ± 0.03 (*P* < 0.05; Mann-Whitney* U* test), suggesting that, in control tissue, DA is helping to maintain a low probability of release. This action has been described for DA D_2_-receptors in terminals of SPNs [20, 37]. In agreement with this result, facilitation increased in the averaged fitted sample (Figure 4(e)) basically by increasing the time constant of recovery (Table 1). Failures also decreased (Figure 4(d) blue). In a representative experiment, the IPSCs variance as a function of IPSC amplitude was clearly different in control versus dopamine depleted synapses and was described by a wider parabola for the dopamine depleted synapse, thus suggesting an increase in the number of release sites or in the available release sites (Figure 4(f) [47]). We further explored these possibilities with modeling techniques.

Similar results were obtained in STB synapses (Figures 4(g)–4(i)) after dopamine loss. Dopamine depletion increased apparent release probability, Pw [47] from (mean ± SEM) 0.31 ± 0.02 to 0.58 ± 0.01 (*P* < 0.05; Mann-Whitney* U* test) and failures became negligible (Figure 4(g) green). However, facilitation decreased in the averaged fitted sample (Figure 4(h)), in agreement with an increase in the depletion time constant (Table 1). Thus, the depressive second part was similar to the depression observed in controls (Figure 4(h)). The IPSC variances as a function of IPSC amplitude were also distributed differently (Figure 4(i)), with a wider parabola for the data from the dopamine depleted tissue suggesting an increase in the number of releasing sites [47]. We also explored this possibility with modeling techniques.

**Figure 5:**
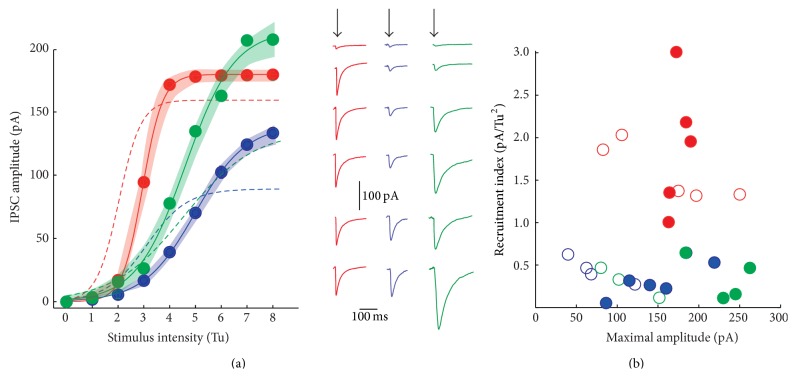
Fits of intensity-amplitude plots in samples of synaptic events with different STSP. (a)* I*-*A* plot of STD (red), STF (blue), and STB (green) sampled averaged synapses; fits ± estimation errors (shaded areas around the fits), also compared with the plots in control conditions from Figure 2 (dashed lines). Differences were not significant for STD synapses but maximal values were significantly different for STF and STB synapses. (b) However, slope factors and recruitment of release sites did not change significantly in these synapses.

The recruitment of synapses and average amplitude changed for connections that exhibited different classes of STSP after DA loss. In average, STD synapses remained very similar to the controls (Table 2 and Figure 5, thick red trace is average of* I*-*A* plot after 6-OHDA, while dashed curve is the control). Mean maximal amplitude increased slightly (Table 2). In addition, the maximal amplitude remained 55% smaller in STF synapses as compared with STD synapses (*P* < 0.05; Kruskal-Wallis and* post hoc* Dunn test; Table 2). In contrast, STB synapses generally displayed larger IPSCS than STD synapses (Figure 5(a)).

In contrast to STD synapses where changes were relatively small, the maximal average amplitude increased for synapses from injured animals in STF (blue) and STB (green) synapses (Figure 4 recordings in left column). In agreement with samples fitting, their amount of maximal inhibition increased by about 64% in STF synapses (*P* < 0.05; Kruskal-Wallis and* post hoc* Dunn test; Table 2) although recruitment parameters did not change significantly, suggesting that they represent probable anatomical disposition of axons and terminals. Although recruitment indicators did not change significantly, there was also an increase in maximal inhibition of about 63% in STB synapses (*P* < 0.05; Kruskal-Wallis and* post hoc* Dunn test; Table 2). In summary, STF and STB synapses, but not STD synapses, decreased their failure rate and increased their release via probable enhancement in release probability and/or the number of release sites available.

To better examine the changes caused by DA loss, a computational model was constructed (see Section 2) to simulate the interaction between depressing and facilitating presynaptic mechanisms. Our simulations reproduce the dynamics displayed by the experimental data. Importantly, the simulations were obtained by assuming that the same two variables, the probability of release and the fraction of occupancy of readily releasable sites, *p* and *x* respectively, represent each and every one of the presynaptic machineries (see Section 2). The state variables in the model evolve with mechanisms that are qualitatively the same but quantitatively different. That is, same functional form different parameters. Note that possible changes in the postsynaptic sites are out of the scope of the present work.

### 3.4. Modeling

**Figure 6:**
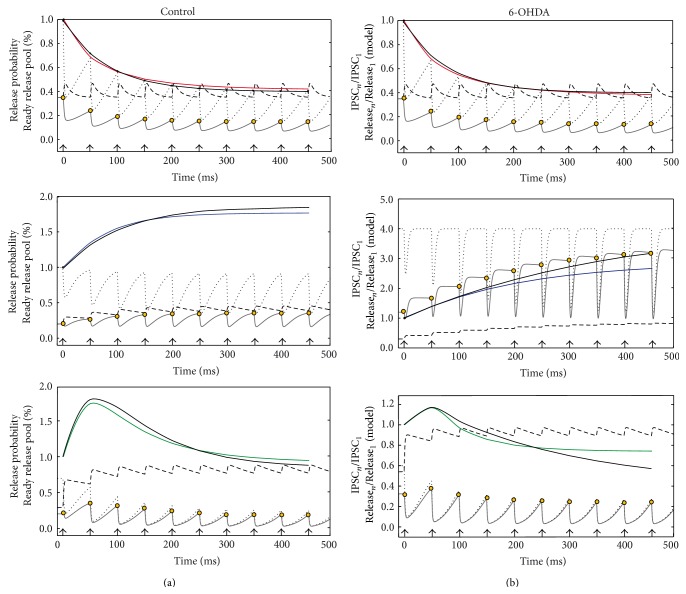
Reproducing experimental results with the proposed new model of short-term synaptic plasticity (see in Section 2 system of equations (3)). (a) Variables of STSP dynamics as a function of a train of stimulus in control conditions: probability of release (*p*, dashed trace), occupancy of the readily releasable pool (*x*, dotted trace). Maximal release (gray trace) after each stimulus is the product *x*(*t*)*∗p*(*t*). Maximal release (yellow circles) for each pulse was normalized to the initial maximal release *x*(0)*p*(0) and plotted in color lines for each synapse (red, blue, and green traces). Experimental traces are in black, to compare with model output (colored). (b) Reproduction of STSP in a 6-OHDA rodent model of Parkinson's disease was less better than the controls; however, it shows the main changes in the release machinery caused by DA depletion (explanation in text).

To model the average behaviors of the different classes of STSP dynamics, we measured the parameters *τ*_Rec_, *τ*_Dep_, *p*(0), *x*(0), and *h* to solve the system of equations (see system of equations (3) in Section 2). Then, we saw how much the model integration approximated the experimental fittings obtained with (1) (Section 2). A train of pulses was given as inputs to trigger synaptic responses (Figure 6). Release probability, *p*(*t*) (dashed line), and occupancy of the ready releasable pool, *x*(*t*) (dotted line), were tracked along time, stimulus by stimulus. The time *x*(0) denotes the initial occupancy of the ready releasable pool, and initial *p*(0) was a value obtained with mean-variance analysis (Figure 4). The amount of neurotransmitter released corresponds to *x*(*t*)*∗p*(*t*) (continuous gray line).

Surprisingly, *p*(*t*) does not change much after the first stimulus in STD synapses (red top) explaining the low probability of failures. However, the occupancy of the readily releasable pool keeps decreasing with each pulse. This causes maximal release (yellow dots on continuous line) to decrease along time, explaining depression. Basically, the same features are preserved in control (left) and DA depleted STD synapses (right). In this case, the model and experimental data were in close agreement (model in red, averaged and fitted experiments, with (1), in black). Model parameters for STD synapses in the control were *τ*_Rec_ = 32 ms, *τ*_Dep_ = 5 ms, *p*(0) = 0.35, *x*(0) = 1, and *h* = 0.1, confirming a faster depletion than recovery. In DA deprivation the parameters were *τ*_Rec_ = 33 ms, *τ*_Dep_ = 6 ms, *p*(0) = 0.35, *x*(0) = 1 and *h* = 0.1, in agreement with the fact that DA deprivation did not significantly affect STD synapses.

In control STF synapses, depletion and recovery rates combine to almost preserve the same occupancy (*x*) of the readily releasable pool during the train of pulses (dotted line). In addition, there was a slight increase in the probability of release (*p*) through time (dashed line). Both factors together produced an increase in release with each stimulus (continuous line with yellow dots). As a consequence, in control conditions, there was a close agreement between the model output and the fit of experimental data (Figure 6(a) middle left). However, to fit the experimental data in 6-OHDA DA depleted tissue, the parameters values changed drastically. Large changes in the initial occupancy value, *x*(*t*), of the readily releasable pool were evident, since it was the only way that the model could approach the experimental data (Figure 6 middle right). After each stimulus the pool was completely and quickly replenished, while *p*(*t*) was only slightly increased. Both factors together produced an increment in release with each stimulus. Nevertheless, further adjustments have to be made in the future to achieve a full convergence with experimental data (blue is the model and black is the experimental fit to the data). The model parameters in control STF synapses were *τ*_Rec_ = 15 ms, *τ*_Dep_ = 60 ms, *p*(0) = 0.2, *x*(0) = 1, and *h* = 0.05, showing that depletion is slower than recovery, as expected. After DA depletion parameters were *τ*_Rec_ = 10 ms, *τ*_Dep_ = 1000 ms, *p*(0) = 0.3, *x*(0) = 4, and *h* = 0.07, suggesting that depletion was much slower and the readily releasable pool was much larger, with little change in *p*(*t*). To conclude, the model predicts that DA deprivation greatly increases the number of available release sites or the size of the readily releasable pool in STF synapses. Whether there are real correlates for these modeling predictions of how dopamine deprivation changes STF synapses is a matter of future experimental and modeling studies.

In control STB synapses, the probability of release (*p*) increased with each stimulus, while occupancy of the readily releasable pool (*x*) decreased, which caused an initial facilitation and posterior depression. There was a close agreement between model and fitted experimental data (Figure 6 bottom left, green is the model and black the experimental data). However, model fits to experimental STB synapses during DA deprivation show a large increase in *p*(*t*). In contrast, there was a slow decrease in occupancy, *x*(*t*) (Figure 6 bottom right; green is model and black experiment). Here again, the model have to be adjusted in the future if the phenomenon is demonstrated experimentally. Model parameters in the control were *τ*_Rec_ = 13 ms, *τ*_Dep_ = 170 ms, *p*(0) = 0.29, *x*(0) = 0.7, and *h* = 0.3, while the same parameters in DA depletion were *τ*_Rec_ = 9.5 ms, *τ*_Dep_ = 200 ms, *p*(0) = 0.54, *x*(0) = 0.7, and *h* = 0.3. In contrast to STF synapses, here the main change was in the initial probability of release, *p*(0), and not the initial occupancy of the readily releasable pool, *x*(*t*).

In summary, the general model proposed here suggests profound changes in the synaptic mechanisms of two classes of GABAergic presynaptic elements impinging on to SPNs after DA deprivation. These changes are different and suggest a series of experiments. More experimental data are necessary to improve the model so that it includes pathological conditions.

To summarize, short-term synaptic plasticity (STSP) of GABAergic inputs making synapses on striatal projection neurons (SPNs) were studied. It was revealed that (1) evoked synaptic trains exhibited three classes of short-term synaptic dynamics: short-term depression (STD), short-term facilitation (STF), and a mixture of facilitation and depression, called biphasic plasticity (STB). In other nuclei where pair recordings between interneurons and projection cells are more easily performed, it has been found that each of these dynamics corresponds to different presynaptic conditions and when experimental conditions are kept equal to different presynaptic neurons [2, 5–7, 11, 14–20]. (2) Our fits and computational simulations show that in STF and STB synapses the release depleting process tends to be slower than in STD synapses, while the probability of release was larger in STD synapses. (3) Intensity-amplitude relationships (*I*-*A* plots) were measured in samples of synaptic recordings exhibiting the three different classes of STSP. It was found that each class of plasticity was associated with a different* I*-*A* plot. It is hard to think how the same presynaptic element may conjoin these multiple combinations of functional parameters. (4) We propose a general mathematical model that reasonably reproduces all these three classes of STSP in control conditions. This model has two main dynamic variables: the probability of release (*p*) and the occupancy of the readily releasable pool (*x*). (5) It has been reported that GABAergic entries on to SPNs suffer functional changes after DA deprivation [27]. Accordingly, we observed that DA depletion differentially affected synapses with different classes of STSP: synapses exhibiting STD were virtually unaffected while synapses exhibiting STF or STB were greatly affected. (6) When the proposed model tried to reproduce the STF dynamics during DA depletion we found indications that the presynaptic machinery was greatly altered so that the model did not exactly reproduce the experimental data. However, the model suggested that the process that was most severely affected was occupancy, *x*(*t*), of the readily releasable pool. There was much more transmitter available to release than in the controls. Further experimental data is needed to both identify this synaptic entry and test whether its availability of transmitter is enhanced. When we tried to reproduce the STB dynamics during DA depletion with our model, we also found evidence of drastic changes in the presynaptic machinery. The model was unable to completely fit all the dynamics displayed by experimental data for the DA depletion experiments. However, the model suggested that the process affected most strongly was an enhanced probability of release, *p*(*t*). A series of new experiments is needed both to identify this type of synaptic entry and to directly test whether its probability of transmitter release is increased. These steps need corroboration before further modification of the model, as each of these variables represents a combination of multiple processes [3, 10].

The number of possible synaptic entries on to projection cells in the striatum is as great as in many other brain nuclei [21, 35, 38–43]. Short-term dynamics between SPNs themselves is well known within the experimental conditions of this work [20, 25] and it is completely different from the three classes of short-term dynamics described here (see Figure 1). Nevertheless, interneurons only encompass 5% of all striatal neurons. The striatum is not a layered or columnar structure. Perhaps because of this, obtaining pair recording between some classes of interneurons and SPNs is a difficult task [21, 42]. Moreover, optogenetic techniques may interfere with the release machinery and change the dynamics in still not well studied ways [23]. Therefore, in the present work, we used short-term dynamics evoked with field stimulation as an indirect way to suspect the presence of a variety of synaptic GABAergic entries on to SPNs. We also used computational modeling to reveal that, in fact, presynaptic elements with diverse release mechanisms, but following the same basic principles, may make synapses on to SPNs, and, furthermore, they could be differentially affected after dopamine deprivation. The consequences that these rearrangements bring about to the Parkinsonian striatal microcircuit are not known. Further, these results raise a number of theoretical and experimental questions about the role played by local interneurons in orchestrating and reorganizing striatal microcircuits that should be addressed in the near future.

## 4. Conclusions

GABAergic synapses on to SPNs could exhibit different types of STSP: short-term depression (STD), short-term facilitation (STF), and a mixture of facilitation and depression, called biphasic plasticity (STB). DA depletion differentially affected synapses that exhibited STF or STB.

## Figures and Tables

**Table 1:** Fitted parameters for synapses exhibiting different types of STSP.

	Depressing synapses		Facilitating synapses		Biphasic synapses	
	Control	6-OHDA	Control	6-OHDA	Control	6-OHDA
*A*	1.15 ± 0.17	**1.41 ± 0.27 **	2.57 ± 0.5	**3.09 ± 1 **	4.4 ± 0.7	**1.9 ± 0.68 **
*τ*_Rec_ (ms)	107.7 ± 37	**120.2 ± 44 **	53.6 ± 15.2	**158.2 ± 28.3 **	62 ± 10.9	**70 ± 20 **
*τ*_Dep_ (ms)	17.2 ± 8.24	**39.3 ± 24 **	29.3 ± 7.6	**37.7 ± 17.2 **	96 ± 5.8	**161 ± 38 **
*B*	−0.5 ± 0.16	**−0.23 ± 0.26 **	2.4 ± 0.62	**4.7 ± 1.6 **	3.2 ± 0.8	**0.41 ± 0.5 **
*C*	0.92 ± 0.14	**0.35 ± 0.6 **	−4.4 ± 2.8	**−18 ± 12 **	−13.2 ± 5.3	**−2 ± 2**

Fitting (1) to sample averages (see Section 2):  IPSC_*n*_/IPSC_1_ = (*A* − *e* ^−*x*/*τ*_Rec_^)*∗*(*e* ^−*x*/*τ*_Dep_^ + *B*) + *C* ± estimation errors.

**Table 2:** Fitting of intensity-amplitude relationships in synapses exhibiting different types of STSP.

	Depressing synapse		Facilitating synapse		Biphasic synapse	
	Control	6-OHDA	Control	6-OHDA	Control	6-OHDA
*A*_max⁡_ (pA)	159 ± 1.5	**180 ± 1.2**	89 ± 6.7	**139 ± 1.5**	135 ± 11.6	**214 ± 9.7**

*k* (Tu/pA)	2.3 ± 0.16	**2.5 ± 0.15**	1.2 ± 0.4	**1 ± 0.03**	0.73 ± 0.12	**1.05 ± 0.14**

*I*_*h*_ (Tu)	2 ± 0.05	**2.9 ± 0.02**	3.1 ± 0.3	**4.9 ± 0.04**	4.5 ± 0.36	**4.6 ± 0.16**

Fitting (2): *A*(*I*) = *A*_max⁡_/1 + *e* ^(−*k*(*I*−*I*_*h*_))^ to the average of IPSC amplitudes of each sample ± estimation error.
